# Supplementary material for: p53 controls choice between apoptotic and non-apoptotic death following DNA damage
Source: bioRxiv. 2023 May 16:2023.01.17.524444. Originally published 2023 Jan 18. Preprint. [Version 3] doi: 10.1101/2023.01.17.524444 (PMC9882237; doi:10.1101/2023.01.17.524444)
Supplement: Supplement 1 [file NIHPP2023.01.17.524444v3-supplement-1.pdf]

bioRxiv preprint doi: https://doi.org/10.1101/2023.01.17.524444; this version posted May 17, 2023. The copyright holder for this preprint (which was not certified by peer review) is the author/funder, who has granted bioRxiv a license to display the preprint in perpetuity. It is made available under aCC-BY 4.0 International license.

SUPPLEMENTAL FIGURE LEGENDS

Supplemental Figure 1: Fractional viability and drug GRADE across p53-proficient and p53-deficient cell lines. Related to Figure 1. (A) Schematic of the FLICK assay and equations for calculating relative viability (RV), fractional viability (FV), and GR values. **(B - C)** Sensitivity of p53 WT and p53 KO cell lines to DNA-damaging chemotherapeutics, as measured by (B) FV or (C) drug GRADE.

Supplemental Figure 2: p53 deletion compromises cell cycle arrest but does not prevent activation of DNA repair or BH3 mimetic-induced apoptosis. Related to Figure 1 and Figure 2. (A) Measurement of cell cycle position using PI staining and the mitotic marker pH-H3. Example for untreated U2OS cells (*left*), and quantification of cell cycle phase from cells treated with nutlin (*right*). **(B)** Live cell counts over time for U2OS and U2OS^{p53KO} cells treated with a sub-lethal dose of etoposide. **(C)** Kinetic western showing the phosphorylation of the DNA damage marker H2AX (phospho-S139) in response to etoposide. Quantification ± SD from 3 experimental replicates (*right*). **(D-F)** Evaluation of inflammatory cell death in U2OS and U2OS^{p53KO} cells. **(D)** Schematic for conditioned media experiment. **(E)** Volcano plot showing the p-values and L2FCs for U2OS cells treated with conditioned media ($\log_2$(U2OS^{p53KO}/U2OS)). **(F)** Pathway-level enrichment for conditioned media, highlighting enrichment for inflammatory signatures in cells treated with media conditioned by U2OS^{p53KO} cells. **(G-H)** Apoptotic priming evaluated using BH3 profiling. **(G)** Basal BH3 profiles in U2OS and U2OS^{p53KO} cells. **(H)** Change in apoptotic priming level following etoposide exposure. **(I)** Activation of apoptotic death in U2OS and U2OS^{p53KO} cells by the BH3 mimetic ABT-199.

Supplemental Figure 3: U2OS and U2OS^{p53KO} treated with cell death inhibitors. Related to Figure 3. (A) U2OS and U2OS^{p53KO} cells treated with single inhibitors for 8 common cell death

bioRxiv preprint doi: https://doi.org/10.1101/2023.01.17.524444; this version posted May 17, 2023. The copyright holder for this preprint (which was not certified by peer review) is the author/funder, who has granted bioRxiv a license to display the preprint in perpetuity. It is made available under aCC-BY 4.0 International license.

pathways. **(B)** U2OS and U2OS^{p53KO} cells treated with higher-order combinations of 5 cell death inhibitors. Heatmap colored by Deviation from Bliss Independence (DBI). Negative DBI values report enhanced lethality, positive DBI reports inhibition of lethality.

Supplemental Figure 4: Chemo-genetic screening analysis strategy and replicate correlation. Related to Figure 3. (A - B) U2OS cells treated with etoposide for 12 days. (A) Live cells were counted to determine the growth defect of each dose. ED = “Effective Dose” (e.g., ED30 = effective dose for 30% reduction in population size after 12 days, compared to untreated). (B) Dead and live cells were counted to determine fractional viability at each dose. **(C)** Analysis schematic for calculating L2FC from chemo-genetic screens. **(D)** Example of correlation between counts for two replicates of the same screen condition. **(E)** Example of correlation between gene-level L2FC values for two screen replicates. z scored L2FC was calculated for each replicate.

Supplemental Figure 5: Validation of rate-based analysis method for chemo-genetic screen. Related to Figure 4. (A) Drug GRADE for U2OS and U2OS^{p53KO} cells treated with etoposide. Dose selected for the CRISPR screen (5 μM) is highlighted. **(B - C)** Schematic for calculation of drug-induced death rate and growth rate from experimentally observed L2FC values. (B) Phase diagram and scatter to highlight one example L2FC that can be produced from multiple combinations of growth and death rate. (C) Calculation of growth rate and inference of the drug-induced death rate. **(D)** Schematic of method used to validate hits from the whole-genome CRISPR screen. **(E)** Validation data generated using FLICK, black = non-targeting sgRNA, blue = targeted gene. **(F)** Phase diagram and scatter plots highlighting validated genes that have a reduced growth rate and are predicted to induce resistance using L2FC. p-values and odds ratios (OR) calculated using a Fishers exact test.

bioRxiv preprint doi: https://doi.org/10.1101/2023.01.17.524444; this version posted May 17, 2023. The copyright holder for this preprint (which was not certified by peer review) is the author/funder, who has granted bioRxiv a license to display the preprint in perpetuity. It is made available under aCC-BY 4.0 International license.

Supplemental Figure 6: Validation of MPT activation in p53 KO cells. Related to Figure 5. **(A)** Gene-level chemo-genetic profiling data for U2OS cells, projected into phase diagram, with OXPHOS genes highlighted. **(B)** as in (A), but for U2OS^{p53KO} cells. **(C)** Phase and SYTOX green images of U2OS cells treated with 10 μM cyclosporin A (CsA). **(D)** Lethal fraction of U2OS and U2OS^{p53KO} cells treated with CsA, zVAD, or CsA+zVAD. **(E)** Cobalt-calcein assay performed on naturally p53-proficient and p53-deficient cell lines treated with 31.6 μM etoposide for 36 hours.

Supplemental Figure 7: Steady state metabolite levels. Related to Figure 7. (A) Metabolite levels shown for vehicle treated U2OS and U2OS^{p53KO} cells at T0. **(B)** Metabolite levels shown for intermediate metabolites involved in glycolysis, PPP, or TCA cycle for U2OS and U2OS^{p53KO}. Data are shown for vehicle and etoposide treated samples at 48 hours. Asterisks report $p<0.05$. ns = not significant. P-values highlighted for metabolites that were not significant at 48 hours but were significantly different at 24 hours. Data are mean ± SD from 3 experimental replicates.

Supplemental Figure 8: Proportional enrichment of glucose-derived metabolites is not altered by the loss of p53. (A) Isotope tracing using $^{13}C_6$-Glucose. Cells were labeled for 8 hours. Fractional enrichment for intermediate metabolites in glycolysis, PPP, and TCA cycle shown for U2OS and U2OS^{p53KO} cells treated with etoposide for 48 hours. **(B)** Data collected as in (A) but analyzed to compare the fractional enrichment of glucose-derived metabolites for upstream and downstream components of glycolysis, PPP, or the TCA cycle. Abbreviations: glucose 6-phosphate (G6P), 2-phosphoglycerate (2PG), ribose 5-phosphate (R5P), uridine monophosphate (UMP).

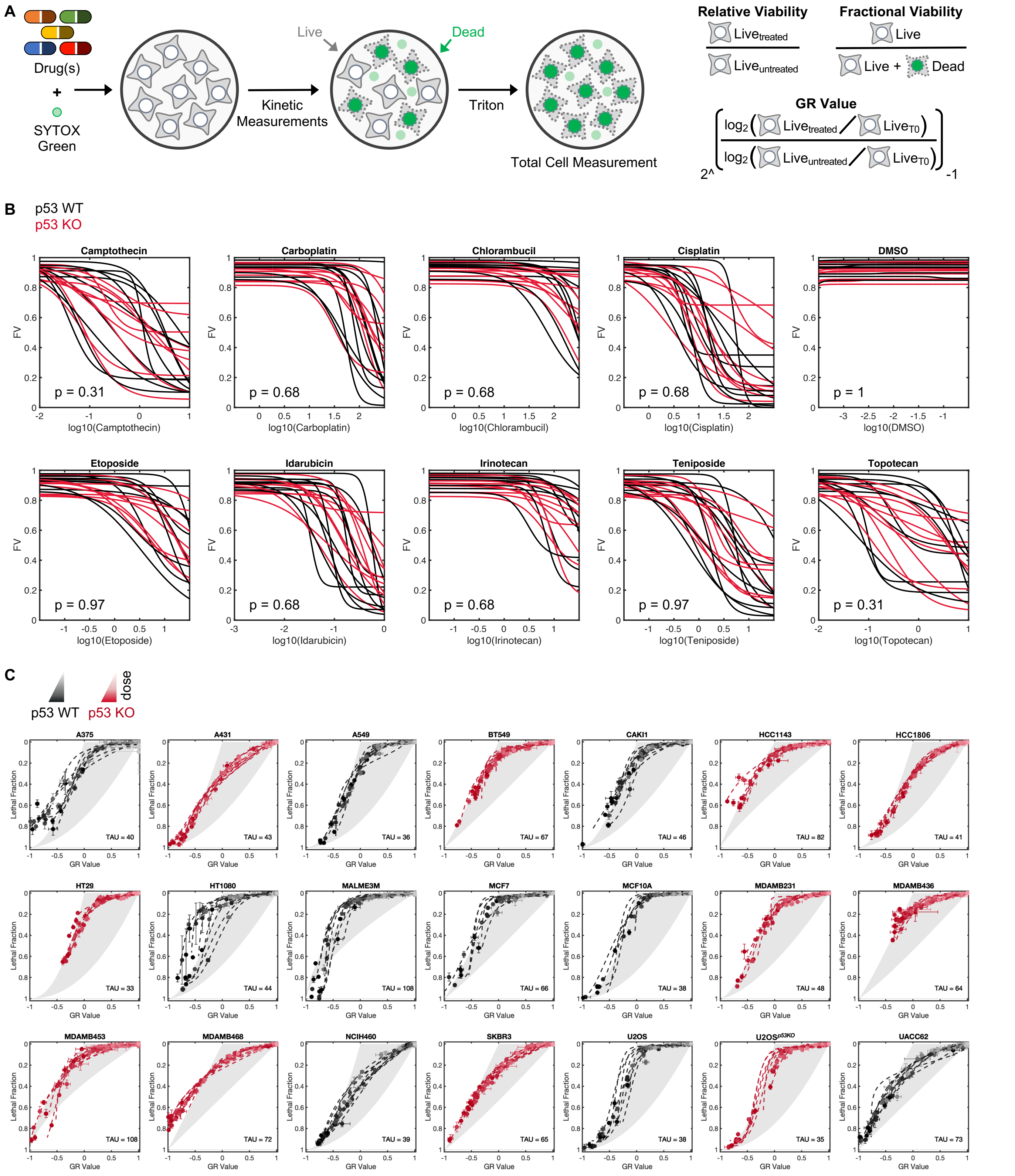

Supplemental Figure 1: Fractional viability and drug GRADE across p53-proficient and p53-deficient cell lines. Related to Figure 1. (A) Schematic of the FLICK assay and equations for calculating relative viability (RV), fractional viability (FV), and GR values. **(B - C)** Sensitivity of p53 WT and p53 KO cell lines to DNA-damaging chemotherapeutics, as measured by (B) FV or (C) drug GRADE.

Honeywell, et al, Supplemental Figure 2

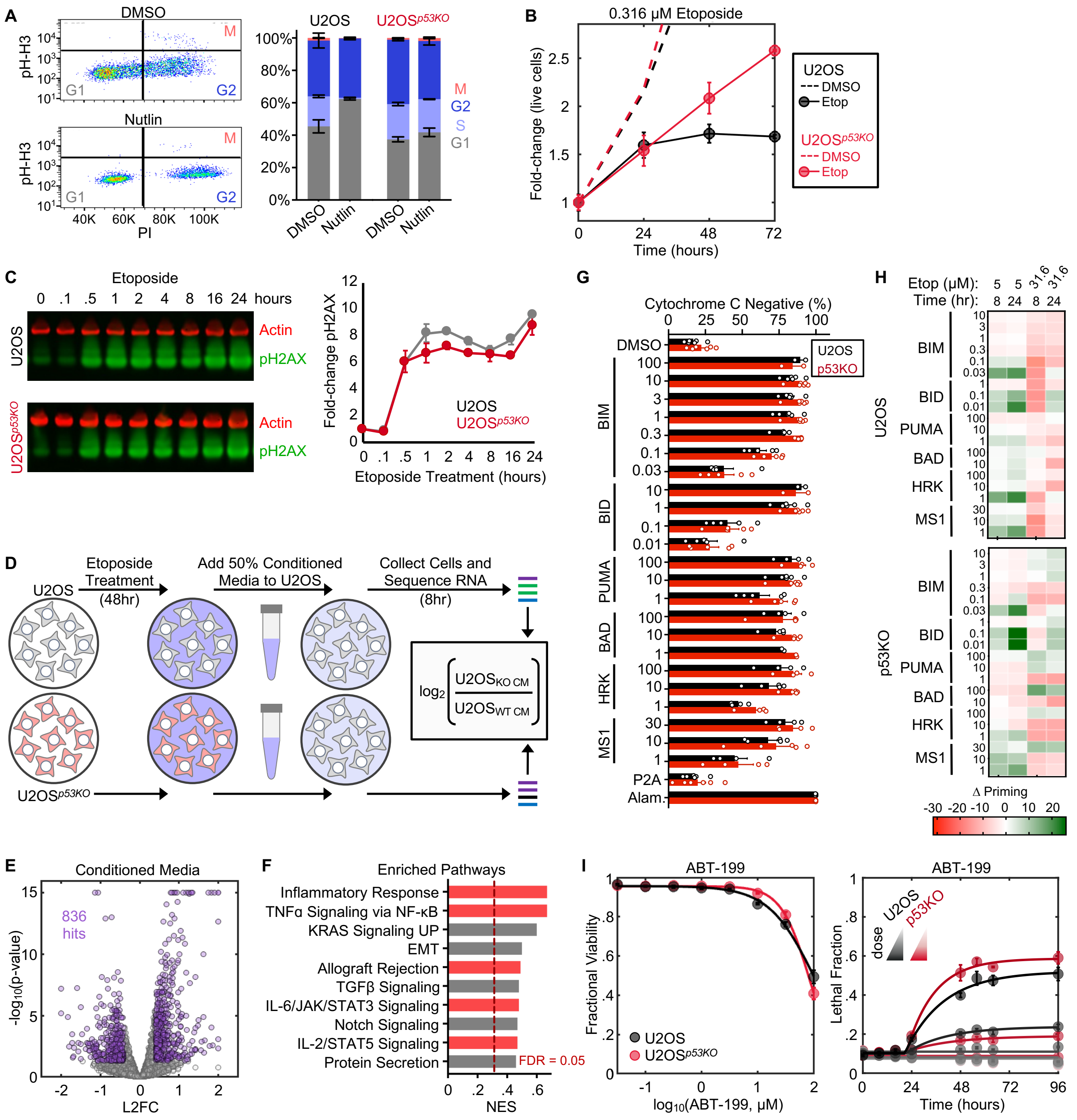

Supplemental Figure 2: p53 deletion compromises cell cycle arrest but does not prevent activation of DNA repair or BH3 mimetic-induced apoptosis. Related to Figure 1 and Figure 2. (A) Measurement of cell cycle position using PI staining and the mitotic marker pH-H3. Example for untreated U2OS cells (*left*), and quantification of cell cycle phase from cells treated with nutlin (*right*). **(B)** Live cell counts over time for U2OS and U2OS^{p53KO} cells treated with a sub-lethal dose of etoposide. **(C)** Kinetic western showing the phosphorylation of the DNA damage marker H2AX (phospho-S139) in response to etoposide. Quantification ± SD from 3 experimental replicates (*right*). **(D-F)** Evaluation of inflammatory cell death in U2OS and U2OS^{p53KO} cells. **(D)** Schematic for conditioned media experiment. **(E)** Volcano plot showing the p-values and L2FCs for U2OS cells treated with conditioned media (log_2(U2OS^{p53KO}/U2OS)). **(F)** Pathway-level enrichment for conditioned media, highlighting enrichment for inflammatory signatures in cells treated with media conditioned by U2OS^{p53KO} cells. **(G-H)** Apoptotic priming evaluated using BH3 profiling. **(G)** Basal BH3 profiles in U2OS and U2OS^{p53KO} cells. **(H)** Change in apoptotic priming level following etoposide exposure. **(I)** Activation of apoptotic death in U2OS and U2OS^{p53KO} cells by the BH3 mimetic ABT-199.

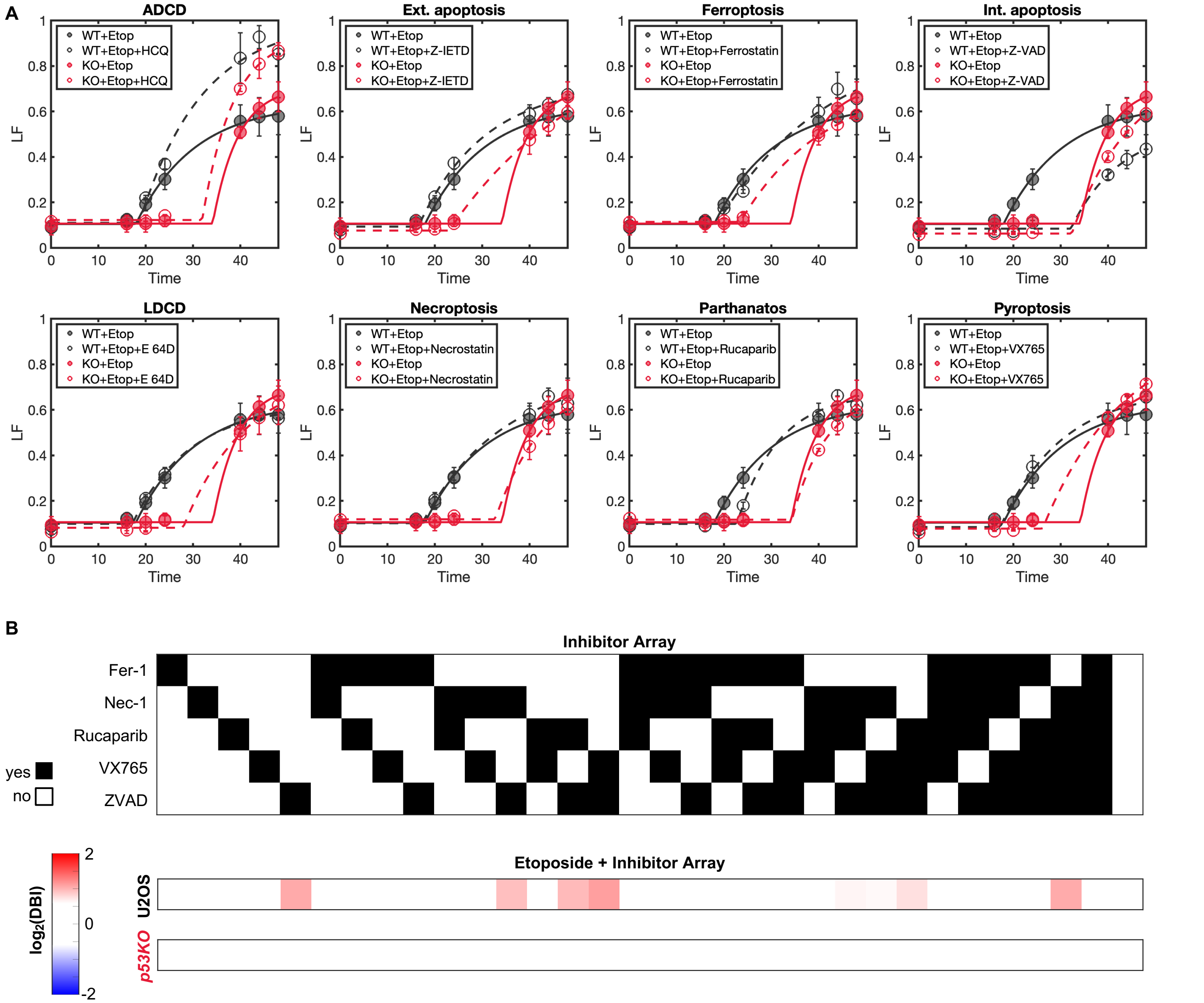

Supplemental Figure 3: U2OS and U2OS^{p53KO} treated with cell death inhibitors. Related to Figure 3. (A) U2OS and U2OS^{p53KO} cells treated with single inhibitors for 8 common cell death pathways. **(B)** U2OS and U2OS^{p53KO} cells treated with higher-order combinations of 5 cell death inhibitors. Heatmap colored by Deviation from Bliss Independence (DBI). Negative DBI values report enhanced lethality, positive DBI reports inhibition of lethality.

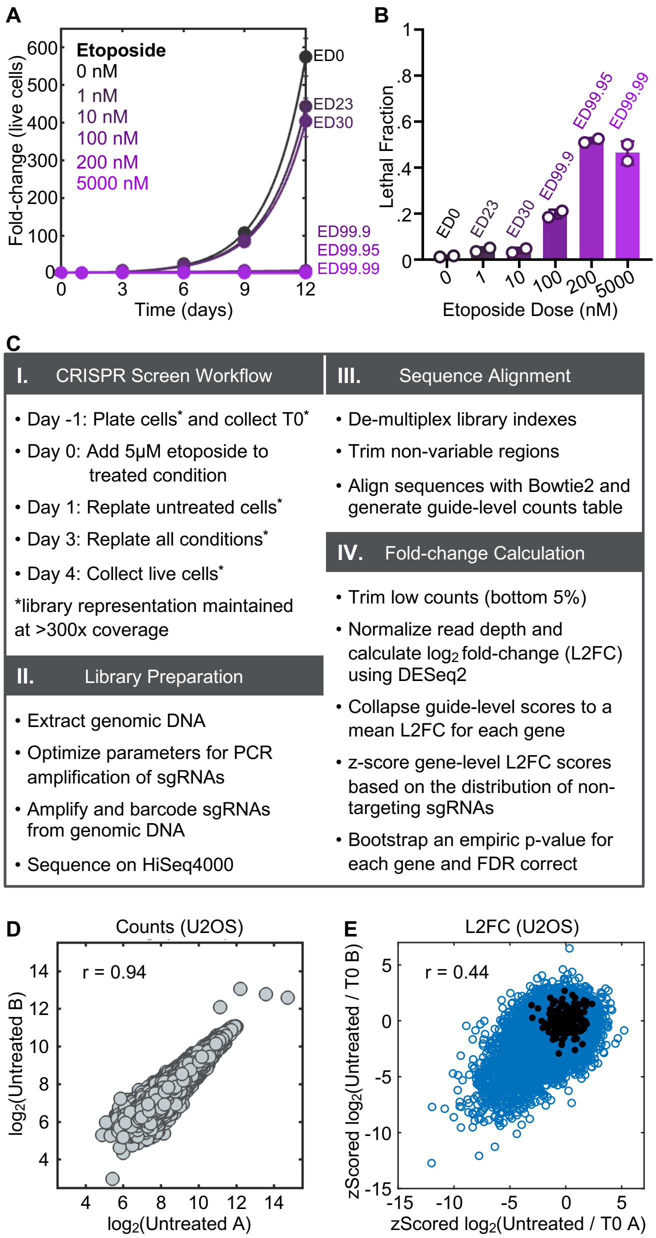

Supplemental Figure 4: Chemo-genetic screening analysis strategy and replicate correlation. Related to Figure 3. (A - B) U2OS cells treated with etoposide for 12 days. (A) Live cells were counted to determine the growth defect of each dose. ED = "Effective Dose" (e.g., ED30 = effective dose for 30% reduction in population size after 12 days, compared to untreated). (B) Dead and live cells were counted to determine fractional viability at each dose. **(C)** Analysis schematic for calculating L2FC from chemo-genetic screens. **(D)** Example of correlation between counts for two replicates of the same screen condition. **(E)** Example of correlation between gene-level L2FC values for two screen replicates. z scored L2FC was calculated for each replicate.

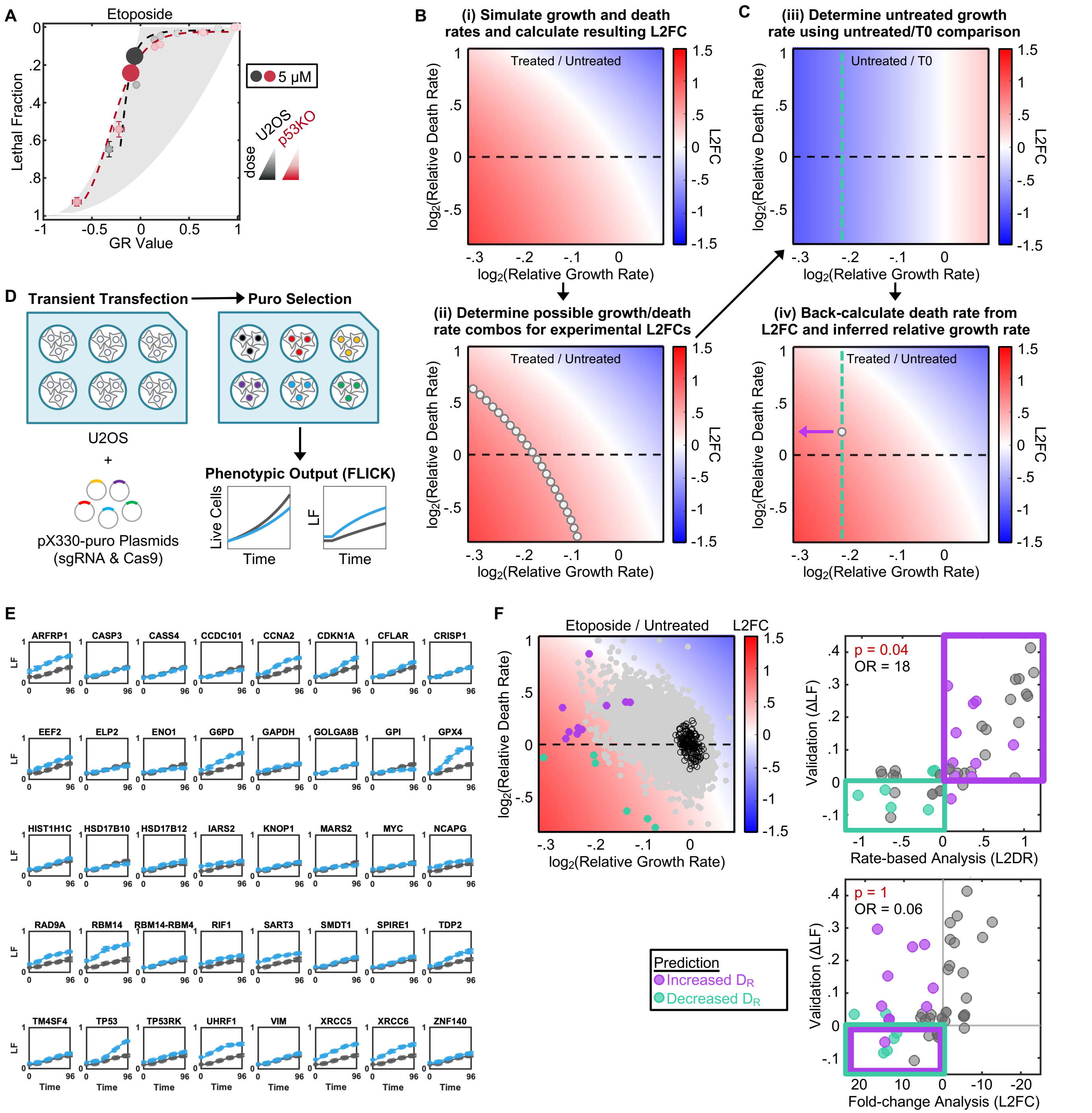

Supplemental Figure 5: Validation of rate-based analysis method for chemo-genetic screen. Related to Figure 4. (A) Drug GRADE for U2OS and U2OS^{p53KO} cells treated with etoposide. Dose selected for the CRISPR screen (5 μM) is highlighted. **(B - C)** Schematic for calculation of drug-induced death rate and growth rate from experimentally observed L2FC values. (B) Phase diagram and scatter to highlight one example L2FC that can be produced from multiple combinations of growth and death rate. (C) Calculation of growth rate and inference of the drug-induced death rate. **(D)** Schematic of method used to validate hits from the whole-genome CRISPR screen. **(E)** Validation data generated using FLICK, black = non-targeting sgRNA, blue = targeted gene. **(F)** Phase diagram and scatter plots highlighting validated genes that have a reduced growth rate and are predicted to induce resistance using L2FC. p-values and odds ratios (OR) calculated using a Fishers exact test.

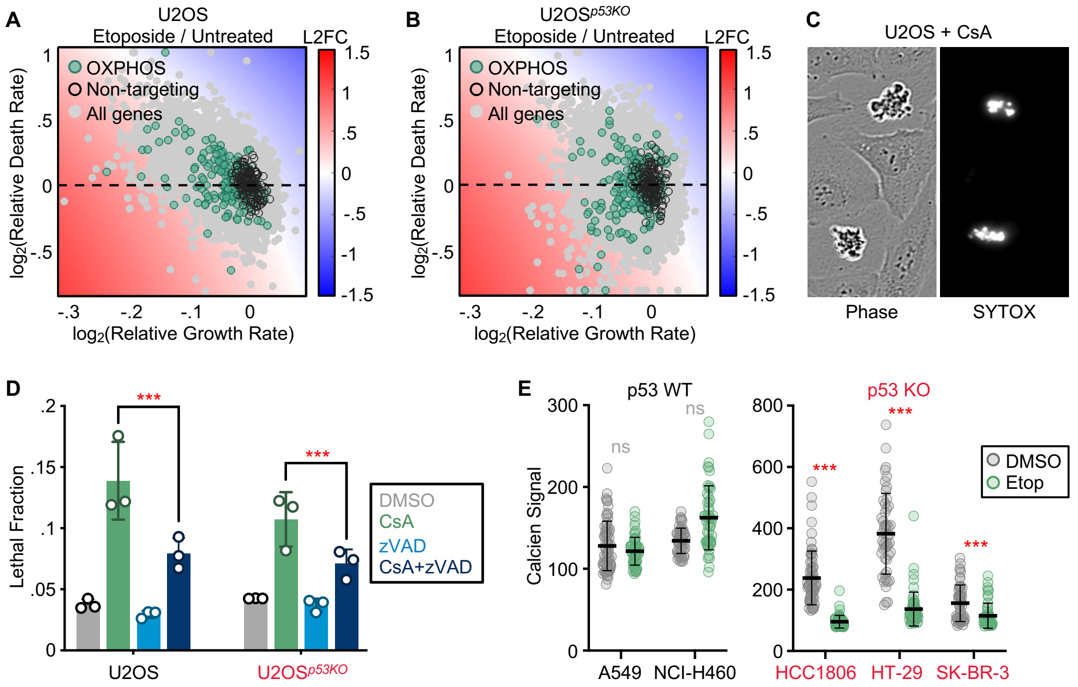

Supplemental Figure 6: Validation of MPT activation in p53 KO cells. Related to Figure 5. (A) Gene-level chemo-genetic profiling data for U2OS cells, projected into phase diagram, with OXPHOS genes highlighted. **(B)** as in (A), but for U2OS^{p53KO} cells. **(C)** Phase and SYTOX green images of U2OS cells treated with 10 μM cyclosporin A (CsA). **(D)** Lethal fraction of U2OS and U2OS^{p53KO} cells treated with CsA, zVAD, or CsA+zVAD. **(E)** Cobalt-calcein assay performed on naturally p53-proficient and p53-deficient cell lines treated with 31.6 μM etoposide for 36 hours.

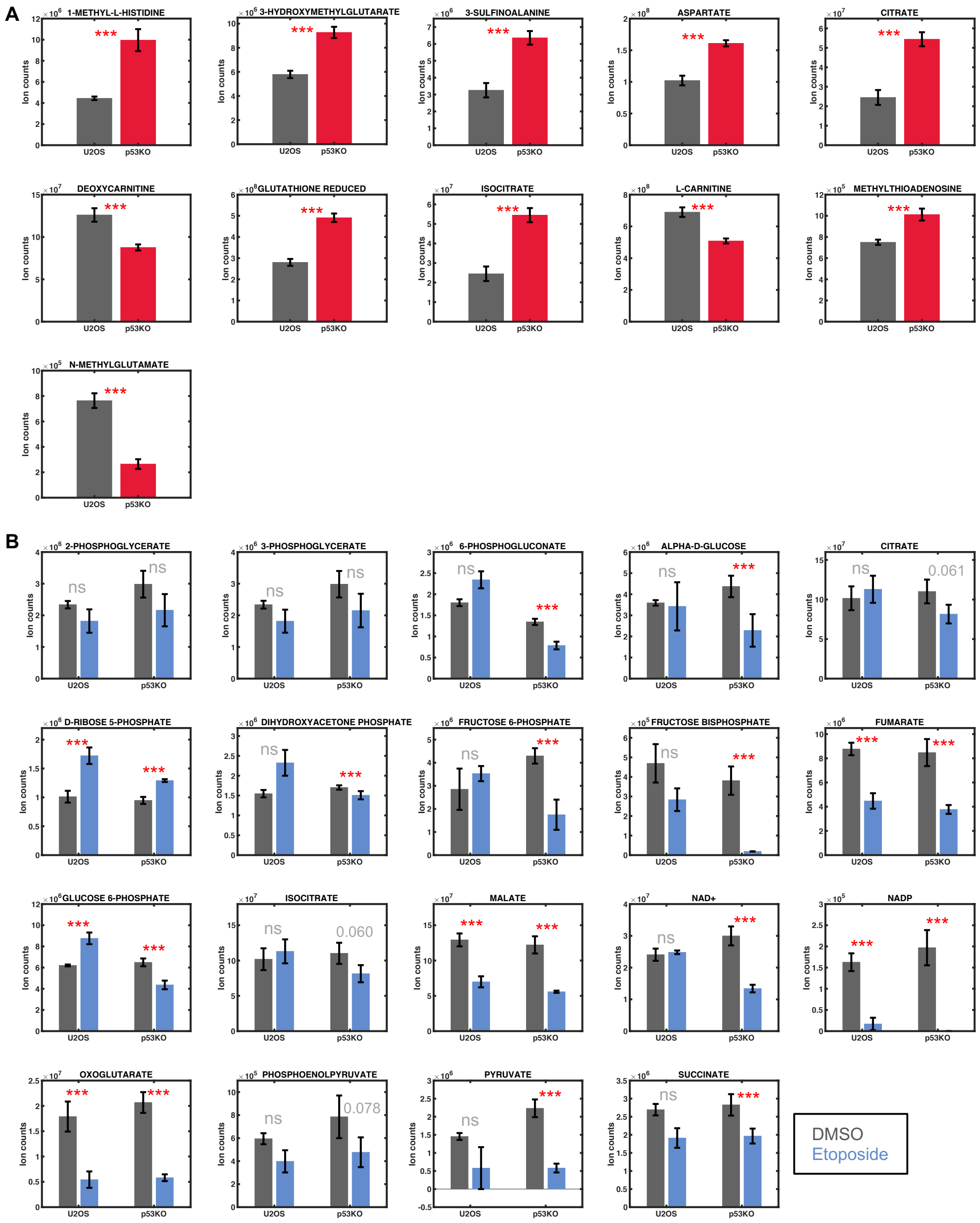

Supplemental Figure 7: Steady state metabolite levels. Related to Figure 7. (A) Metabolite levels shown for vehicle treated U2OS and U2OS^{p53KO} cells at T0. **(B)** Metabolite levels shown for intermediate metabolites involved in glycolysis, PPP, or TCA cycle for U2OS and U2OS^{p53KO}. Data are shown for vehicle and etoposide treated samples at 48 hours. Asterisks report p<0.05. ns = not significant. P-values highlighted for metabolites that were not significant at 48 hours but were significantly different at 24 hours. Data are mean ± SD from 3 experimental replicates.

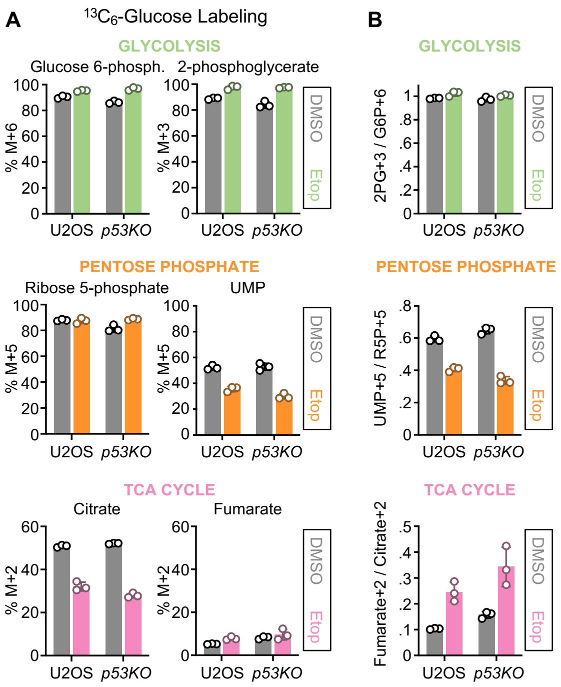

Supplemental Figure 8: Proportional enrichment of glucose-derived metabolites is not altered by the loss of p53. (A) Isotope tracing using $^{13}C_6$-Glucose. Cells were labeled for 8 hours. Fractional enrichment for intermediate metabolites in glycolysis, PPP, and TCA cycle shown for U2OS and U2OS^{p53KO} cells treated with etoposide for 48 hours. **(B)** Data collected as in (A) but analyzed to compare the fractional enrichment of glucose-derived metabolites for upstream and downstream components of glycolysis, PPP, or the TCA cycle. Abbreviations: glucose 6-phosphate (G6P), 2-phosphoglycerate (2PG), ribose 5-phosphate (R5P), uridine monophosphate (UMP).
